# Performance and Psychometric Properties of Novel Brief Assessments for Depression in Children and Adolescents

**DOI:** 10.1016/j.jaacop.2024.05.002

**Published:** 2024-05-27

**Authors:** Paul E. Croarkin, Paul A. Nakonezny, David W. Morris, A. John Rush, Betsy D. Kennard, Graham J. Emslie

**Affiliations:** a Mayo Clinic, Rochester, Minnesota; b UT Southwestern Medical Center in Dallas, Texas; c Duke-National University of Singapore, Singapore, Singapore, and Duke University School of Medicine, Durham, North Carolina

**Keywords:** brief rating scale, major depressive disorder, measurement-based care, psychometric, rating scale

## Abstract

**Objective:**

Current rating scales for depressive symptom severity in pediatric patients do not meet the needs of contemporary clinical practice and research. This study sought to evaluate relative performance and psychometric properties of the 5-item Brief Children’s Depression Rating Scale—Revised (BCDRS-R5), as well as the clinician-rated and self-report versions of the 5-item Very Quick Inventory of Depressive Symptomatology (VQIDS-A5-C and VQIDS-A5-SR, respectively).

**Method:**

This study examined a sample of 165 outpatients aged 8 to 17 years with major depressive disorder who were treated openly with fluoxetine for 6 weeks from a prior National Institute of Mental Health (NIMH)–funded study. We examined the internal consistency, scale dimensionality, relative performance in detecting remission and response, and sensitivity to change of each 5-item scale.

**Results:**

All 3 brief scales had good-to-excellent internal consistency. Cronbach coefficient α values were 0.687 to 0.795 at baseline and 0.766 to 0.844 at week 6. Principal component analysis suggested a 1-factor solution for each scale. The BCDRS-R5 demonstrated a greater degree of accuracy in identifying response and remission compared to the VQIDS-A5-C and VQIDS-A5-SR. The scales were sensitive to change in symptom severity over 6 weeks of acute treatment with fluoxetine.

**Conclusion:**

Three novel, brief scales assessing depressive symptom severity in pediatric patients showed similar performance and sensitivity to change in symptom severity over 6 weeks of acute treatment when compared with longer, standard scales.

**Clinical trial registration information:**

Sequential Treatment of Pediatric MDD to Increase Remission and Prevent Relapse; https://clinicaltrials.gov/study/NCT00612313.

## Plain language summary

Existing assessment tools for depressive symptom severity in children and adolescents have limitations and do not meet the needs of contemporary families, clinical practice, or research efforts. The purpose of the current study, using a sample of youth with depression, was to evaluate the psychometric properties of the 5-item Brief Children's Depression Rating Scale–Revised (BCDRS-R5) as well as the 5-item Very Quick Inventory of Depressive Symptomatology self-report and clinician-rated versions (VQIDS-A5-SR/VQIDS-A5-C), compare their relative performance, and define clinically-relevant depressive symptom severity thresholds for remission. These novel, brief scales had similar performance as longer, standard scales and show promise for future use in clinical practice and research efforts.

Current rating scales for assessing depressive symptom severity in children and adolescents have limitations in the context of clinical trials, for the implementation of measurement-based care, and in clinical translational research.^1^, ^2^, ^3^ An ideal rating scale would be acceptable to families, busy clinicians, and researchers. This ideal scale would be efficient to administer, valid, reliable, and sensitive to change compared with existing instruments.^4^, ^5^, ^6^, ^7^ Digital platforms and decentralized care models will soon revolutionize clinical practice, but standard rating scales of depressive symptom severity have not evolved for use in this novel clinical and research space.^8^^,^^9^

The Patient Health Questionnaire—9 (PHQ-9)^10^ and related adaptations such as the Patient Health Questionnaire—9 Modified (PHQ-9M) are now standard screening and assessment tools for clinical practice.^11^ In general, the PHQ-9M is acceptable for clinical practice and patients.^12^ For more than general screening, however, the PHQ-9M has substantial psychometric limitations, particularly for the assessment of treatment outcomes.^12^ Conversely, the Children’s Depression Rating Scale—Revised (CDRS-R17) is the gold standard instrument for clinical trials in children and adolescents with major depressive disorder (MDD).^13^, ^14^, ^15^ The CDRS-R17 is seldom administered in clinical practice settings because it has a substantial time burden for patients and clinicians.^12^ The Quick Inventory of Depressive Symptoms (QIDS) has adolescent-, parent-, and clinician-rated adaptations for the assessment of depressive symptoms in youth.^16^ The QIDS instruments also have a problematic time burden for patients, clinical practice, and future digital practice.^12^ Assessments of fluctuating depressive symptoms such as insomnia and appetite are often difficult to interpret and unreliable.^17^ Ideal future assessment tools of depressive symptom severity will meet collective needs for acceptability, efficiency, validity, and reliability.^7^^,^^8^^,^^18^ Brief assessment tools would facilitate translational bridges between clinical care and research efforts for pediatric patients with MDD.^17^^,^^19^^,^^20^

Recent efforts have examined and validated brief versions of the QIDS for adults with MDD^19^^,^^20^ and the CDRS-R17 in youth with MDD.^17^ Prior efforts also suggested that a 6-item adaptation of the Hamilton Rating Scale for Depression (HRSD) accounted for the majority of the variance in depressive symptom severity outcome offered by the standard 17-item HRSD.^21^ The current study sought to adapt, expand, and innovate these efforts for the assessment of MDD in children and adolescents. A brief version of the CDRS-R in particular would have utility for clinical practice and future interventional research.^17^

This study focused on a rigorously characterized sample of children and adolescents with MDD undergoing open treatment with fluoxetine. We examined the psychometric properties of the 5-item Brief Children’s Depression Rating Scale—Revised (BCDRS-R5) as well as the 5-item Very Quick Inventory of Depressive Symptomatology self-report and clinician-rated versions (VQIDS-A5-SR and VQIDS-A5-C), compared their relative performance, and defined clinically relevant depressive symptom severity thresholds for remission. We hypothesized that BCDRS-R5, VQIDS-A5-C, and VQIDS-A5-SR would have acceptable performance and psychometric properties in comparison to the longer, standard versions of each respective instrument.

## Method

This study used data obtained from the acute treatment phase of a National Institute of Mental Health (NIMH)–funded single-site, randomized relapse prevention continuation trial in youth with MDD.^22^^,^^23^ A total of 200 participants aged 8 to 17 years with a primary diagnosis of nonpsychotic major depressive disorder were enrolled and treated openly with fluoxetine for 6 weeks (acute treatment phase), and then those with an adequate response (defined as a reduction of 50% or more on the CDRS-R17) were randomly assigned to receive continued medication management alone or continued medication management plus cognitive–behavioral therapy for an additional 6 months (continuation phase).^22^^,^^23^ The study was approved by the local Institutional Review Board. All participants and their parents provided written consent and assent. A detailed description of the full methodology and outcomes of the NIMH-funded Relapse Prevention Trial (RPT) has been previously reported.^23^

### Current Study Participants

The participants of the current study are a subset of participants from the above-mentioned acute treatment phase of the RPT who had both a CDRS-R17 and a Quick Inventory of Depressive Symptomatology patient self-report (QIDS-A17-SR) and clinician-rated (QIDS-A17-C) assessment at baseline. Of the original 200 participants enrolled in the acute-phase treatment, 165 youth had the CDRS-R17, QIDS-A17-SR, and the QIDS-A17-C rating scales at baseline and thus were included in this study.^23^

### Measures

The study outcomes as well as schedule of assessments for the RPT have been previously reported.^23^ For the current study, however, we used outcomes of symptom severity collected at baseline and at each medication management visit (weeks 2, 4, and 6) during the acute treatment phase of the RPT.^23^ Symptom severity was assessed using the CDRS-R17,^13^^,^^15^ the QIDS-A17-SR, and the QIDS-A17-C.^16^ The QIDS-A17-C, however, was assessed only at baseline and week 6 during the acute-phase treatment.

The CDRS-R17 consists of 17 items. The first 14 items of the CDRS-R17 are rated from the patient’s and parent’s responses, and the last 3 items are from the rater’s observations (facial affect, speech, and hypoactivity). The CDRS-R17 items are rated on 5-point (items 4, 5, and 16) or 7-point (items 1–3, 6–15, and 17) Likert-type scales. The clinician provides an overall summary score on the basis of interviews and scores of the parent and child, and the total score ranges from 17 to 113 (with a higher score representing greater depressive symptom severity).

The QIDS-A17-SR and QIDS-A17-C both have 3 components with adolescent and parent self-report scores and a composite report completed by the interviewer. The QIDS-A17-SR is a self-report screening instrument, and the QIDS-A17-C is a clinician-rated instrument. Both instruments consist of 17 items, with each item scored from 0 to 3; the total score is calculated on the basis of a scoring system in which the highest scores from questions 1 through 4, questions 5 and 6, questions 7 through 10, and questions 16 and 17 are added to scores from questions 11 through 15. The total score on the QIDS-A17-SR and QIDS-A17-C ranges from 0 to 27, with higher scores representing greater severity of depressive symptoms.

This study also used the 5-item Very Quick Inventory of Depressive Symptomatology self-report and clinician-rated versions (VQIDS-A5-SR and VQIDS-A5-C; total score range 0-15)^19^^,^^20^ and the 5-item Brief Children’s Depression Rating Scale—Revised version (BCDRS-R5; total score range 5-35).^17^ The VQIDS-A5-C and VQIDS-A5-SR were derived from the QIDS-A17-C and QIDS-A17-SR, respectively, and included items 5 (feeling sad), 12 (self-outlook), 14 (general interest), 15 (energy level), and 16 (psychomotor slowing).^19^^,^^20^ The BCDRS-R5 was derived from the CDRS-R17 and included items 2 (difficulty having fun), 3 (social withdrawal), 10 (low self-esteem), 11 (depressed feelings), and 15 (depressed facial affect).^17^ Details and psychometric properties on each of the scales can be found elsewhere.^17^^,^^19^^,^^20^ We also note that our own item response theory (IRT) methods, which were implemented in this sample using the graded response model by PROC IRT procedures in SAS software, confirmed the 5-items for each of the VQIDS-A5-C, VQIDS-A5-SR, and BCDRS-R5 used in this study (Tables S1 and S2 online).

Remission status was defined based on the gold standard CDRS-R17 total score of ≤28^1^^,^^24^^,^^25^ at each medication management visit (weeks 2, 4, and 6) during the acute treatment phase of the RPT. Categories of remission (for the other scales used in this study) that emerged from the ROC analysis were compared against this gold standard definition of remission.

Treatment response, irrespective of remission, was operationally defined as a decrease of at least 50% in total score (symptom severity) on each respective scale from baseline to weeks 2, 4, and 6, respectively.

### Statistical Analysis

The mean-item total correlations, the scale mean and SD, as well as the internal consistency (Cronbach coefficient α) for each scale were determined by using classical test theory (CTT) analysis. Dimensionality of the VQIDS-A5-C, VQIDS-A5-SR, and BCDRS-R5 was defined by principal component analysis (PCA) using varimax rotation. The CTT analysis was applied at baseline and week 6.

A receiver operating characteristic (ROC) analysis was conducted to determine the optimal cut point for each scale (based on the Youden index) in discriminating remission status at the completion of 6 weeks of acute treatment based on the gold standard definition of remission of CDRS-R17 total ≤28.^22^^,^^23^ The area under the curve (AUC), sensitivity, and specificity were also reported for each optimal cut point.

The percentage of participants who were remitters vs non-remitters and responders vs non-responders from baseline to weeks 2, 4, and 6 were reported. Benefit categories, which were applied to all scales, included treatment response irrespective of remission (≥50% reduction in symptom severity from baseline) and categories of remission that emerged from the ROC analysis. The strength of agreement between the various pairs of measures (CDRS-R17, BCDRS-R5, QIDS-A17-C, VQIDS-A5-C, QIDS-A17-SR, and VQIDS-A5-SR) was assessed by the kappa statistic, positive predictive value (PPV), negative predictive value (NPV), sensitivity, specificity, classification accuracy rate, and classification error rate for remission vs no remission and response vs no response.

Finally, the comparative sensitivity of the various scales (measures) to change in symptom severity over 6 weeks of acute treatment with fluoxetine was assessed by computing the percent change from baseline to weeks 2, 4, and 6. A dependent-samples *t* test was used to test for differences in the mean percent change at each time period. The Cohen *d* (which accounted for the within-subjects correlation of the paired values) was also calculated and interpreted as the effect size estimator for the relative magnitude of change.

All statistical analyses were performed using SAS software, version 9.4 (SAS Institute, Inc) and MedCalc for Windows, version 20.218 (MedCalc Software). The procedures in MedCalc were used to conduct the ROC analyses. The level of significance for all tests was set at an α level of 0.05 (2-tailed).

## Results

### Participant Characteristics

The evaluable sample in this study consisted of 165 participants from the acute treatment phase of the RPT, which included 51.52% female adolescents (n = 85) and 75.76% adolescents (n = 125), with a mean age of 13.55 ± 2.59 years (range, 8-17 years). Mean CDRS-R17 total, QIDS-A17-C, and QIDS-A17-SR scores at baseline were 56.51 ± 10.93, 15.97 ± 3.91, and 11.89 ± 5.62, respectively, which consistently reflected moderate symptom severity. Mean CDRS-R17 total, QIDS-A17-C, and QIDS-A17-SR scores following 6 weeks of acute treatment were 33.01 ± 9.39, 7.69 ± 4.27, and 6.62 ± 5.64, respectively. About 32% of youth had a CDRS-R17 total score ≤28 (ie, were in remission) following 6 weeks of acute treatment. Participant characteristics are reported in Table 1.

**Table 1:** Demographic and Clinical Characteristics of the Study Sample (N = 165)

Participant characteristics	Value
Demographics	
Age, y, mean ± SD (range 8-17)	13.55 ± 2.59
Female, n (%)	85 (51.52)
Children (aged 8-11 y), n (%)	40 (24.24)
Adolescents (aged 12 -17 y), n (%)	125 (75.76)
Race, n (%)	
White	138 (83.64)
African American	17 (10.30)
Asian	03 (1.82)
Multiracial	07 (4.24)
Ethnicity, n (%)	
Hispanic	52 (31.52)
Non-Hispanic	113 (68.48)
Clinical characteristics	
CDRS-R17 total at baseline, mean ± SD	56.51 ± 10.93
CDRS-R17 total at week 6, mean ± SD	33.01 ± 9.39
BCDRS-R5 at baseline, mean ± SD	19.52 ± 4.16
BCDRS-R5 at week 6, mean ± SD	10.96 ± 3.74
QIDS-A17-C total at baseline, mean ± SD	15.97 ± 3.91
QIDS-A17-C total at week 6, mean ± SD	7.69 ± 4.27
VQIDS-A5-C at baseline, mean ± SD	8.68 ± 2.75
VQIDS-A5-C at week 6, mean ± SD	3.78 ± 2.71
QIDS-A17-SR total at baseline, mean ± SD	11.89 ± 5.62
QIDS-A17-SR total at week 6, mean ± SD	6.62 ± 5.64
VQIDS-A5-SR at baseline, mean ± SD	5.67 ± 3.62
VQIDS-A5-SR at week 6, mean ± SD	3.01 ± 3.35

Note: BCDRS-R5 = Brief Children’s Depression Rating Scale—Revised; CDRS-R17 = Children’s Depression Rating Scale—Revised; QIDS-A17-C = Quick Inventory of Depressive Symptomatology, clinician-rated; QIDS-A17-SR = Quick Inventory of Depressive Symptomatology patient self-report; VQIDS-A5-C = Very Quick Inventory of Depressive Symptomatology, clinician-rated; VQIDS-A5-SR = Very Quick Inventory of Depressive Symptomatology, patient self-rated.

### Internal Consistency and Scale Dimensionality

The internal consistency (Cronbach coefficient α) of the VQIDS-A5-C, VQIDS-A5-SR, and BCDRS-R5 ranged from 0.687 to 0.795 at baseline and from 0.766 to 0.844 at week 6. Corresponding Cronbach α values for QIDS-A17-C, QIDS-A17-SR, and CDRS-R17 at baseline were 0.756, 0.821, and 0.791, respectively. Corresponding Cronbach α values at week 6 were 0.807, 0.884, and 0.867, respectively. The PCA suggested a 1-factor solution for the VQIDS-A5-C, VQIDS-A5-SR, and BCDRS-R5. The percentage of total variance explained by each of the sole principal components ranged from 45.31% to 55.13% at baseline and from 52.84% to 61.92% at week 6. The CTT results are reported in Tables S1 and S2 (available online).

### ROC Analysis

The ROC analysis determined the optimal cut point for each scale in discriminating remission status at the completion of 6 weeks of acute treatment based on the gold standard CDRS-R17 total ≤28.^22^^,^^23^ As a result of this ROC analysis, the remission categories (cut points) were defined as a score of ≤2 on the VQIDS-A5-SR (AUC 0.773; sensitivity: 83.33%; specificity: 59.09%) and VQIDS-A5-C (AUC 0.852; sensitivity: 75.61%; specificity: 82.14%), ≤8 on the BCDRS-R5 (AUC 0.921; sensitivity: 69.00%; specificity: 95.50%), and ≤5 on the QIDS-A17-SR (AUC 0.828; sensitivity: 71.43%; specificity: 82.95%) and QIDS-A17-C (AUC 0.907; sensitivity: 78.00%; specificity: 88.10%).

### Comparisons for Categories of Benefit

Benefit categories included treatment response irrespective of remission (≥50% reduction in symptom severity from baseline) and remission that emerged from the ROC analysis. Figure S1A and S1B, available online, show the percentage of participants who were remitters vs non-remitters and responders vs non-responders from baseline to weeks 2, 4, and 6. Tables 2 through 4 show the strength of agreement for the various pairs of measures from baseline to weeks 2, 4, and 6, respectively (the full version of Table 2, Table 3, Table 4, with all indices, is available online as Tables S3-S5, available online, whereas the concise version, with only the classification accuracy and error rates, is reported in this article). We note that QIDS-A17-C and VQIDS-A5-C were assessed only at baseline and week 6. From baseline to week 2, the BCDRS-R5 vs CDRS-R17 demonstrated a high degree of agreement (accuracy) in detecting remission vs no remission (correct classification rate 96.24%) and response vs no response (correct classification rate 90.98%). Also, from baseline to week 2, the VQIDS-A5-SR vs QIDS-A17-SR had excellent agreement in detecting remission vs no remission (correct classification rate 89.31%). However, there was only a modest level of agreement of the QIDS-A17-SR/VQIDS-A5-SR with the CDRS-R17 in detecting remission vs no remission (correct classification rate ranged from 60.90% to 71.43%) and response vs no response (correct classification rate ranged from 62.60% to 79.39%) from baseline to week 2.

**Table 2:** Classification Accuracy and Error Rates Between the Pairs of Scales in Detecting Remission and Response (Baseline to Week 2)

Index of agreement	RPT (n = 133)							
	Baseline to wk 2							
	CDRS-R17 BCDRS-R5	CDRS-R17 BCDRS-R5	QIDS-A17-SR VQIDS-A5-SR	QIDS-A17-SR VQIDS-A5-SR	CDRS-R17 VQIDS-A5-SR	CDRS-R17 VQIDS-A5-SR	CDRS-R17 QIDS-A17-SR	CDRS-R17 QIDS-A17-SR
	Remission	Response	Remission	Response	Remission	Response	Remission	Response
Accuracy or correct classification rate, %	96.24	90.98	89.31	77.10	60.90	62.60	71.43	79.39
Error rate or misclassification rate, %	3.76	9.02	10.69	22.90	39.10	37.40	28.57	20.61

Note: The Remission category was defined as a score of ≤2 on the VQIDS-A5-SR, ≤8 on the BCDRS-R5, ≤5 on the QIDS-A17-SR, and ≤28 on the CDRS-R17. The Response category was defined as a ≥50% reduction in symptom severity from baseline to wk 2. The overall classification accuracy rate corresponds to the proportion of observations that were correctly classified: (true positive + true negative)/(true positive + false positive + true negative + false negative). The classification error rate was defined as the proportion of observations that were misclassified; error rate = 100 – accuracy. BCDRS-R5 = Brief Children’s Depression Rating Scale—Revised; CDRS-R17 = Children’s Depression Rating Scale—Revised; QIDS-A17-C = Quick Inventory of Depressive Symptomatology, clinician-rated; QIDS-A17-SR = Quick Inventory of Depressive Symptomatology patient self-rated; RPT = relapse prevention trial; VQIDS-A5-C = Very Quick Inventory of Depressive Symptomatology, clinician-rated; VQIDS-A5-SR = Very Quick Inventory of Depressive Symptomatology, patient self-rated.

**Table 3:** Classification Accuracy and Error Rates Between the Pairs of Scales in Detecting Remission and Response (Baseline to Week 4)

Index of agreement	RPT (n = 117)							
	Baseline to wk 4							
	CDRS-R17 BCDRS-R5	CDRS-R17 BCDRS-R5	QIDS-A17-SR VQIDS-A5-SR	QIDS-A17-SR VQIDS-A5-SR	CDRS-R17 VQIDS-A5-SR	CDRS-R17 VQIDS-A5-SR	CDRS-R17 QIDS-A17-SR	CDRS-R17 QIDS-A17-SR
	Remission	Response	Remission	Response	Remission	Response	Remission	Response
Accuracy or correct classification rate, %	92.31	90.60	91.45	83.76	66.67	69.83	71.80	73.28
Error rate or misclassification rate, %	7.69	9.40	8.56	16.24	33.33	30.17	28.20	26.72

Note: The Remission category was defined as a score of ≤2 on the VQIDS-A5-SR, ≤8 on the BCDRS-R5, ≤5 on the QIDS-A17-SR, and ≤28 on the CDRS-R17. The Response category was defined as a ≥50% reduction in symptom severity from baseline to wk 4. The overall classification accuracy rate corresponds to the proportion of observations that were correctly classified: (true positive + true negative)/(true positive + false positive + true negative + false negative). The classification error rate was defined as the proportion of observations that were misclassified; error rate = 100 – accuracy. BCDRS-R5 = Brief Children’s Depression Rating Scale—Revised; CDRS-R17 = Children’s Depression Rating Scale—Revised; RPT = relapse prevention trial; QIDS-A17-C = Quick Inventory of Depressive Symptomatology, clinician-rated; QIDS-A17-SR = Quick Inventory of Depressive Symptomatology patient self-rated; VQIDS-A5-C = Very Quick Inventory of Depressive Symptomatology, clinician-rated; VQIDS-A5-SR = Very Quick Inventory of Depressive Symptomatology, patient self-rated.

**Table 4:** Classification Accuracy and Error Rates Between the Pairs of Scales in Detecting Remission and Response (Baseline to Week 6)

Index of agreement	RPT (n = 130)								RPT (n = 125)					
	Baseline to wk 6													
	CDRS-R17 BCDRS-R5	CDRS-R17 BCDRS-R5	QIDS-A17-SR VQIDS-A5-SR	QIDS-A17-SR VQIDS-A5-SR	CDRS-R17 VQIDS-A5-SR	CDRS-R17 VQIDS-A5-SR	CDRS-R17 QIDS-A17-SR	CDRS-R17 QIDS-A17-SR	QIDS-A17-C VQIDS-A5-C	QIDS-A17-C VQIDS-A5-C	CDRS-R17 VQIDS-A5-C	CDRS-R17 VQIDS-A5-C	CDRS-R17 QIDS-A17-C	CDRS-R17 QIDS-A17-C
	Remission	Response	Remission	Response	Remission	Response	Remission	Response	Remission	Response	Remission	Response	Remission	Response
Accuracy or correct classification rate, %	86.92	89.23	89.23	83.08	66.92	72.09	70.00	65.90	83.87	87.90	79.84	84.68	84.68	82.26
Error rate or misclassification rate, %	13.08	10.77	10.77	16.92	33.08	27.91	30.00	34.10	16.13	12.10	20.16	15.32	15.32	17.74

Note: The Remission category was defined as a score of ≤2 on the VQIDS-A5-SR and VQIDS-A5-C, ≤8 on the BCDRS-R5, ≤5 on the QIDS-A17-SR and QIDS-A17-C, and ≤28 on the CDRS-R17. The Response category was defined as a ≥50% reduction in symptom severity from baseline to wk 6. The overall classification accuracy rate corresponds to the proportion of observations that were correctly classified: (true positive + true negative)/(true positive + false positive + true negative + false negative). The classification error rate was defined as the proportion of observations that were misclassified; error rate = 100 – accuracy. BCDRS-R5 = Brief Children’s Depression Rating Scale—Revised; CDRS-R17 = Children’s Depression Rating Scale—Revised; QIDS-A17-C = Quick Inventory of Depressive Symptomatology, clinician-rated; QIDS-A17-SR = Quick Inventory of Depressive Symptomatology patient self-rated; RPT = relapse prevention trial; VQIDS-A5-C = Very Quick Inventory of Depressive Symptomatology, clinician-rated; VQIDS-A5-SR = Very Quick Inventory of Depressive Symptomatology, patient self-rated.

From baseline to weeks 4 and 6, the VQIDS-A5-SR vs QIDS-A17-SR and the BCDRS-R5 vs CDRS-R17 demonstrated a high degree of accuracy in detecting remission vs no remission (correct classification rate ranged from 86.92% to 92.31%) and response vs no response (correct classification rate ranged from 83.08% to 83.76%). Also, at week 6, the VQIDS-A5-C vs QIDS-A17-C demonstrated a moderate to high degree of accuracy in detecting remission vs no remission (correct classification rate: 83.87%) and response vs no response (correct classification rate: 87.90%). Moreover, at week 6, the QIDS-A17-C/VQIDS-A5-C vs CDRS-R17 demonstrated a reasonable degree of accuracy in detecting remission vs no remission (correct classification rate range: 79.84% to 84.68%) and response vs no response (correct classification rate range: 82.26% to 84.68%).

### Sensitivity to Change Over 6 Weeks of Acute Treatment

The comparative sensitivity of the various scales to change over the 6 weeks of acute treatment was evaluated by the percent change from baseline to weeks 2, 4, and 6. These results, as shown in Table 5, albeit similar, revealed that the relative magnitude of the sensitivity of the various scales to change in depressive symptom severity (as evaluated by the Cohen *d*) was greatest for CDRS-R17 followed by the BCDRS-R5, QIDS-A17-C, VQIDS-A5-C, QIDS-A17-SR, and VQIDS-A5-SR. Clearly the clinician ratings have larger effect sizes here, as they embodied less variability (ie, had smaller SDs) and thus more narrow confidence intervals than the self-report ratings.

**Table 5:** Mean Percent Change Over 6 Weeks of Acute Treatment Among the Various Scales

Clinical outcome	Baseline to wk 2			Baseline to wk 4			Baseline to wk 6		
	Mean % change (SD)	95% CI	*d*	Mean % change (SD)	95% CI	*d*	Mean % change (SD)	95% CI	*d*
QIDS-A17-SR	–24.01 (38.07)	–30.66 to –17.34	0.44	–31.48 (55.13)	–41.66 to –21.29	0.46	–43.95 (37.39)	–50.51 to –37.38	1.09
VQIDS-A5-SR	–32.15 (47.53)	–40.89 to –23.41	0.48	–39.99 (46.44)	–48.98 to –31.01	0.63	–48.99 (44.99)	–57.23 to –40.75	0.98
CDRS-R17	–21.79 (14.31)	–24.24 to –19.33	1.33	–33.97 (17.18)	–37.11 to –30.82	2.07	–42.85 (14.86)	–45.43 to –40.27	3.26
BCDRS-R5	–21.65 (16.84)	–24.54 to –18.76	1.19	–35.61 (19.65)	–39.21 to –32.01	1.90	–45.51 (16.95)	–48.44 to –42.56	2.89
QIDS-A17-C	--			--			–50.75 (27.08)	–55.57 to –45.95	2.37
VQIDS-A5-C	--			--			–54.96 (32.66)	–60.77 to –49.16	2.09

Note: Dependent-samples *t* test was used to test for differences in the mean percent change at each time period. All *p* values were <.0001. QIDS-C17 and VQIDS-C5 were assessed only at baseline and week 6. d = Cohen d effect size estimator (which accounted for the within-subjects correlation of the paired values). BCDRS-R5 = Brief Children’s Depression Rating Scale—Revised; CDRS-R17 = Children’s Depression Rating Scale—Revised; QIDS-A17-C = Quick Inventory of Depressive Symptomatology, clinician-rated; QIDS-A17-SR = Quick Inventory of Depressive Symptomatology patient self-rated; VQIDS-A5-C = Very Quick Inventory of Depressive Symptomatology, clinician-rated; VQIDS-A5-SR = Very Quick Inventory of Depressive Symptomatology, patient self-rated.

## Discussion

This study examined the psychometric properties and relative performance of the VQIDS-A5-C, VQIDS-A5-SR, and BCDRS-R5 with the goal of innovating novel, brief assessments for depressive symptom severity in children and adolescents with MDD for clinical practice and research settings. The VQIDS-A5-C, VQIDS-A5-SR, and BCDRS-R5 demonstrated good-to-excellent internal consistency. Principal component analysis supported a 1-factor solution for the VQIDS-A5-C, VQIDS-A5-SR, and BCDRS-R5. The Remission categories (defined as a score of ≤2 on the VQIDS-A5-SR and VQIDS-A5-C, ≤8 on the BCDRS-R5, and ≤5 on the QIDS-A17-SR and QIDS-A17-C) were supported by ROC analysis findings and are consistent with those reported by Rush *et al.*, who examined the VQIDS and QIDS in adults with major depressive disorder.^16^^,^^20^ The BCDRS-R5, VQIDS-A5-SR, and VQIDS-A5-C all demonstrated a high degree of agreement (ie, accuracy) in detecting response and remission when compared to longer, standard rating scales.^12,22,23.^ The QIDS-A17-SR/VQIDS-A5-SR had modest agreement with the CDRS-R17 in detecting clinical outcomes. Conversely, the clinician-rated versions QIDS scales, the QIDS-A17-C/VQIDS-A5-C had good agreement with the CDRS-R17. This is not unexpected, as prior work consistently suggests that self-reported and clinician-rated measures of depressive symptom severity often do not have ideal agreement, underscoring the importance of collecting both categories of rating scales in clinical practice and research.^12^^,^^20^

Although a prior study identified 5 CDRS-R17 items reflecting disease severity, the present study was the first psychometric validation of this brief scale (BCDRS-R5).^17^ Prior studies have also focused on the VQIDS-A5-SR/VQIDS-A5-C in adults with MDD, but to our knowledge, no prior study has examined the psychometric properties and relative performance of the VQIDS-A5-SR/VQIDS-A5-C in children and adolescents with MDD.^19^^,^^20^

Our findings support a 1-factor solution for the VQIDS-A5-C, VQIDS-A5-SR, and BCDRS-R5, whereas prior exploratory factor analysis studies of the CDRS-R17 have identified 2 to 5 factors.^2^ A unifactorial rating scale for adolescents with depression has the potential to address critical unmet needs, as prior work suggests that unifactorial rating scales that are more sensitive to change in symptoms are more likely to differentiate clinical effects of an antidepressant from placebo in clinical trials.^26^, ^27^, ^28^, ^29^, ^30^, ^31^ The BCDRS-R5 may have distinct advantages as a unitary clinical rating scale for depressive symptom severity in youth, as it provides a reliable framework for resolving a common challenge with discrepancies in clinician and self-report ratings.^4^^,^^32^, ^33^, ^34^ The BCDRS-R5 may prove to be a useful clinical tool for assessing treatment effects during brief primary care clinic visits, virtual visits, and with digital therapeutic interventions. In a clinical context, implementation of the BCDRS-R5 would be a noteworthy innovation for practice, with potential improvements integrating parent and child reports in addition to assessing symptom severity changes with treatment.

Stallwood *et al.*^2^ presented concerns that the CDRS-R may not be a suitable assessment tool for depressive symptoms in adolescents in the context of lacking or low evidence for content validity, cross-cultural validity, and reliablity. The present findings do not address these knowledge gaps and critiques. The present study is an important foundation for future efforts to develop adequate outcome measurement instruments for the assessment of depressive symptom severity in children and adolescents.^2^ Accurate, appropriate, and culturally valid outcome measurements are a critical unmet need for our field that is consistently highlighted by failed clinical trials.^2^^,^^3^^,^^7^ Future outcome measurements (scales) must also demonstrate feasibility, validity, and reliability in decentralized studies, as well as application with digital platforms. Future studies of the VQIDS-A5-C, VQIDS-A5-SR, and BCDRS-R5 should also assess content validity, cross-cultural validity, and measurement error in adolescents.

Limitations of the current study include the use of a single sample in the context of a historical clinical trial using pen-and-paper assessments. Although the data were collected in an urban area in the context of a clinical trial, the sample was relatively homogeneous (83.64% White, non-Hispanic patients). This aspect of the sample limits the generalizability of the present findings. As noted above, the present study also used the CDRS-R17 as gold standard measurement. Despite extensive prior use of the CDRS-R17 in clinical trials, there are unanswered questions regarding its utility as a measurement instrument for depressive symptom severity in adolescents.^2^ The present findings do not necessarily establish scale dimensionality. Moreover, depressive disorders and symptoms are heterogeneous. The 5-item brief scales will not capture specific profiles. For example, differential findings with initial, middle, and terminal insomnia and hypersomnia have implications for the identification of melancholic and atypical depressive disorders. The 5-item scales will not have utility in characterizing differences in the clinical syndrome of depression. There are no population norms for the VQIDS-A5-C, VQIDS-A5-SR, and BCDRS-R5. All three 5-item brief scales should be administered as separate scales concurrently with the respective full-version scale, and future research should then evaluate the relative performance and psychometric properties each brief scale. Replication studies with larger, diverse samples using tablet, smartphone, or wearable ecological momentary assessment data collection platforms should also be conducted.^8^^,^^9^ Finally, it is important to recognize that the abridged scales used in the present study do not have items assessing suicidal ideation or suicidal intent. Concurrent clinical or research assessments must always include direct narrative questions and validated scales that assess suicidal thoughts and behaviors.^35^ Notwithstanding these limitations, this initial work suggests that novel, brief assessment tools such as the VQIDS-A5-C, VQIDS-A5-SR, and BCDRS-R5 have promise for addressing unmet needs in contemporary clinical practice and research settings that are focused on the treatment of children and adolescents with depression.

In conclusion, this study demonstrates the feasibility, validity, internal consistency, accuracy of outcome identification, and sensitivity to change of novel, brief assessments of depressive symptom severity in children and adolescents. The BCDRS-R5, VQIDS-A5-C, and VQIDS-A5-SR have promise as clinical and research assessment instruments.

## CRediT authorship contribution statement

**Paul E. Croarkin:** Writing – review & editing, Writing – original draft, Methodology, Investigation, Formal analysis, Data curation, Conceptualization. **Paul A. Nakonezny:** Writing – review & editing, Writing – original draft, Methodology, Investigation, Formal analysis, Data curation, Conceptualization. **David W. Morris:** Writing – review & editing, Writing – original draft, Methodology, Conceptualization. **A. John Rush:** Writing – review & editing, Writing – original draft, Methodology, Formal analysis, Conceptualization. **Betsy D. Kennard:** Writing – review & editing, Writing – original draft, Methodology, Investigation, Formal analysis, Data curation, Conceptualization. **Graham J. Emslie:** Writing – review & editing, Writing – original draft, Methodology, Investigation, Formal analysis, Data curation, Conceptualization.
