# Supplementary material for: Performance and Psychometric Properties of Novel Brief Assessments for Depression in Children and Adolescents
Source: JAACAP Open. 2024 May 27;3(2):335–43. doi: 10.1016/j.jaacop.2024.05.002 (PMC12166918; doi:10.1016/j.jaacop.2024.05.002)
Supplement: Tables S1 and S2 [file mmc1.docx]

Table 1. Baseline VQIDS-A_5_-C, VQIDS-A_5_-SR, and BCDRS-R_5_ Internal Consistency and Scale Dimensionality and IRT

|  |  |  |  |  | **Factor 1 Pattern^a^** | | **IRT Parameter Estimate^b^** |
| --- | --- | --- | --- | --- | --- | --- | --- |
| **VQIDS-A_5_-C Scale Items at**  **Baseline (N=165)** |  | **Mean** | ***SD*** | ***r*_it_** | **Loadings** | **Communalities** | **Slope** |
| Sad Mood |  | 2.113 | 0.803 | 0.606 | 0.773 | 0.598 | 2.23 |
| Self-Outlook |  | 1.729 | 0.970 | 0.405 | 0.582 | 0.339 | 1.14 |
| Involvement |  | 1.581 | 0.952 | 0.641 | 0.815 | 0.664 | 2.38 |
| Energy |  | 1.615 | 0.906 | 0.548 | 0.733 | 0.538 | 1.62 |
| Psychomotor Slowing |  | 0.936 | 0.868 | 0.549 | 0.723 | 0.523 | 1.63 |
|  |  |  |  |  |  |  |  |
| Variance Explained by Factor | 53.29% |  |  |  |  |  |  |
| Cronbach's Coefficient α | 0.773 |  |  |  |  |  |  |
|  |  |  |  |  |  |  |  |
| **VQIDS-A_5_-SR Scale Items at**  **Baseline (N=165)** |  | **Mean** | ***SD*** | ***r*_it_** | **Loadings** | **Communalities** | **Slope** |
| Sad Mood |  | 1.545 | 0.959 | 0.612 | 0.771 | 0.595 | 2.29 |
| Self-Outlook |  | 1.121 | 1.022 | 0.530 | 0.701 | 0.491 | 1.82 |
| Involvement |  | 0.903 | 1.001 | 0.588 | 0.752 | 0.565 | 2.10 |
| Energy |  | 1.284 | 1.022 | 0.618 | 0.779 | 0.607 | 1.82 |
| Psychomotor Slowing |  | 0.769 | 0.873 | 0.532 | 0.705 | 0.497 | 1.71 |
|  |  |  |  |  |  |  |  |
| Variance Explained by Factor | 55.13% |  |  |  |  |  |  |
| Cronbach's Coefficient α | 0.795 |  |  |  |  |  |  |
|  |  |  |  |  |  |  |  |
| **BCDRS-R_5_ Scale Items at**  **Baseline (N=165)** |  | **Mean** | ***SD*** | ***r*_it_** | **Loadings** | **Communalities** | **Slope** |
| Difficulty Having Fun |  | 4.175 | 1.347 | 0.603 | 0.824 | 0.679 | 1.50 |
| Social Withdrawal |  | 3.636 | 1.405 | 0.538 | 0.783 | 0.613 | 1.41 |
| Low Self-Esteem |  | 4.345 | 1.262 | 0.336 | 0.533 | 0.284 | 0.88 |
| Depressed Feelings |  | 4.769 | 1.145 | 0.502 | 0.725 | 0.526 | 2.13 |
| Depressed Facial Affect |  | 2.600 | 1.058 | 0.242 | 0.402 | 0.161 | 0.72 |
|  |  |  |  |  |  |  |  |
| Variance Explained by Factor | 45.31% |  |  |  |  |  |  |
| Cronbach's Coefficient α | 0.687 |  |  |  |  |  |  |

^a^ Extraction method was from a Principal Components Analysis (rotation not possible with 1 retained factor).

^b^ Item Response Theory (IRT) methods were implemented using the graded response model.

Table 2. VQIDS-C5, VQIDS-SR5, and BCDRS-R5 Internal Consistency and Scale Dimensionality and IRT at Week 6

|  |  |  |  |  | **Factor 1 Pattern^a^** | | **IRT Parameter Estimate^b^** |
| --- | --- | --- | --- | --- | --- | --- | --- |
| **VQIDS-A_5_-C Scale Items at**  **Week 6 (N=125)** |  | **Mean** | ***SD*** | ***r*_it_** | **Loadings** | **Slope** | **Slope** |
| Sad Mood |  | 0.984 | 0.718 | 0.576 | 0.749 | 0.561 | 2.14 |
| Self-Outlook |  | 1.024 | 0.911 | 0.494 | 0.674 | 0.454 | 1.49 |
| Involvement |  | 0.608 | 0.717 | 0.439 | 0.619 | 0.384 | 1.07 |
| Energy |  | 0.792 | 0.743 | 0.606 | 0.791 | 0.626 | 1.71 |
| Psychomotor Slowing |  | 0.376 | 0.655 | 0.601 | 0.784 | 0.615 | 2.18 |
|  |  |  |  |  |  |  |  |
| Variance Explained by Factor | 52.84% |  |  |  |  |  |  |
| Cronbach's Coefficient α | 0.766 |  |  |  |  |  |  |
|  |  |  |  |  |  |  |  |
| **VQIDS-A_5_-SR Scale Items at**  **Week 6 (N=130)** |  | **Mean** | ***SD*** | ***r*_it_** | **Loadings** | **Communalities** | **Slope** |
| Sad Mood |  | 0.816 | 0.892 | 0.709 | 0.829 | 0.687 | 3.10 |
| Self-Outlook |  | 0.603 | 0.942 | 0.611 | 0.751 | 0.565 | 2.58 |
| Involvement |  | 0.465 | 0.726 | 0.588 | 0.730 | 0.534 | 2.17 |
| Energy |  | 0.664 | 0.873 | 0.701 | 0.826 | 0.682 | 2.91 |
| Psychomotor Slowing |  | 0.465 | 0.816 | 0.654 | 0.791 | 0.626 | 2.67 |
|  |  |  |  |  |  |  |  |
| Variance Explained by Factor | 61.92% |  |  |  |  |  |  |
| Cronbach's Coefficient α | 0.844 |  |  |  |  |  |  |
|  |  |  |  |  |  |  |  |
| **BCDRS-R_5_ Scale Items at**  **Week 6 (N=130)** |  | **Mean** | ***SD*** | ***r*_it_** | **Loadings** | **Communalities** | **Slope** |
| Difficulty Having Fun |  | 2.138 | 1.001 | 0.665 | 0.816 | 0.666 | 1.98 |
| Social Withdrawal |  | 2.076 | 1.053 | 0.648 | 0.802 | 0.643 | 1.65 |
| Low Self-Esteem |  | 2.800 | 1.088 | 0.454 | 0.621 | 0.386 | 1.28 |
| Depressed Feelings |  | 2.453 | 1.086 | 0.634 | 0.785 | 0.617 | 3.24 |
| Depressed Facial Affect |  | 1.492 | 0.717 | 0.594 | 0.747 | 0.559 | 1.61 |
|  |  |  |  |  |  |  |  |
| Variance Explained by Factor | 57.47% |  |  |  |  |  |  |
| Cronbach's Coefficient α | 0.804 |  |  |  |  |  |  |

^a^ Extraction method was from a Principal Components Analysis (rotation not possible with 1 retained factor

^b^ Item Response Theory (IRT) methods were implemented using the graded response model.
