# Supplementary material for: Performance and Psychometric Properties of Novel Brief Assessments for Depression in Children and Adolescents
Source: JAACAP Open. 2024 May 27;3(2):335–43. doi: 10.1016/j.jaacop.2024.05.002 (PMC12166918; doi:10.1016/j.jaacop.2024.05.002)
Supplement: Figure S1 [file mmc5.docx]

Figure S1A. Percentage of participants who were remitters vs. non-remitters

from baseline to weeks 2, 4, and 6. The Remission category was defined as a score of ≤2 on

the VQIDS-A5-SR and VQIDS-A5-C, ≤8 on the BCDRS-R5, ≤5 on the QIDS-A17-SR and QIDS-A17-C,

and ≤28 on the CDRS-R17. The Response category was defined as a ≥ 50% reduction in symptom

severity from baseline to weeks 2, 4, and 6.

Figure S1B. Percentage of participants who were responders vs. non-responders

from baseline to weeks 2, 4, and 6. The Response category was defined as a ≥ 50% reduction

in symptom severity from baseline to weeks 2, 4, and 6.
